# Diagnosis of Urinary Tract Infections by Urine Flow Cytometry: Adjusted Cut-Off Values in Different Clinical Presentations

**DOI:** 10.1155/2019/5853486

**Published:** 2019-03-03

**Authors:** Sabine K. Schuh, Ruth Seidenberg, Spyridon Arampatzis, Alexander B. Leichtle, Wolf E. Hautz, Aristomenis K. Exadaktylos, Clyde B. Schechter, Martin Müller

**Affiliations:** ^1^Department of Emergency Medicine, Inselspital, Bern University Hospital, University of Bern, Bern, Switzerland; ^2^Department of Anesthesiology, Inselspital, Bern University Hospital, University of Bern, Bern, Switzerland; ^3^Department of Nephrology, Hypertension and Clinical Pharmacology, Inselspital, Bern University Hospital, University of Bern, 3010 Bern, Switzerland; ^4^Department of Clinical Chemistry and Insel Data Science Center (IDSC), Inselspital, Bern University Hospital, University of Bern, Bern, Switzerland; ^5^Department of Family & Social Medicine & Department of Epidemiology and Population Health, Albert Einstein College of Medicine, Bronx, New York, USA

## Abstract

**Background:**

Bacterium and leucocyte counts in urine can be measured by urine flow cytometry (UFC). They are used to predict significant bacterial growth in urine culture and to diagnose infections of the urinary tract. However, little information is available on appropriate UFC cut-off values for bacterium and leucocyte counts in specific clinical presentations.

**Objective:**

To develop, validate, and evaluate adapted cut-off values that result in a high negative predictive value for significant bacterial growth in urine culture in common clinical presentation subgroups.

**Methods:**

This is a single center, retrospective, observational study with data from patients of the emergency department of Bern University Hospital, Switzerland, with suspected infections of the urinary tract. The patients presented with different symptoms, and urine culture and urine flow cytometry were performed. For different clinical presentations, the patients were grouped by (i) age (>65 years), (ii) sex, (iii) clinical symptoms (e.g., fever or dysuria), and (iv) comorbidities such as diabetes and immunosuppression. For each group, cut-off values were developed, validated, and analyzed using different strategies, i.e., linear discriminant analysis (LDA) and Youden's index, and were compared with known cut-offs and cut-offs optimized for sensitivity.

**Results:**

613 patients were included in the study. Significant bacterial growth in urine culture depended on clinical presentation and ranged from 32.3% in male patients to 61.5% in patients with urinary frequency. In all clinical presentations, the predictive accuracy of UFC leucocyte and UFC bacterium counts was good for significant bacterial growth in urine culture (AUC ≥ 0.88). The adapted LDA_95_ equations did not exhibit consistently high sensitivity. However, the in-house cut-offs (test positive if UFC leucocytes > 17/*μ*L or UFC bacteria > 125/*μ*L) were highly sensitive (>90%). In female, younger, and dysuric patients, even higher cut-offs for UFC leucocytes (169/*μ*L, 169/*μ*L, and 205/*μ*L) exhibited high sensitivity. Specificity was insufficient (<0.9) for all tested cut-offs.

**Conclusions:**

For various clinical presentations, significant bacterial growth in urine culture can be excluded if flow cytometry measurements give a bacterial count of ≤125/*μ*L or a leucocyte count of ≤17/*μ*L. In female patients, dysuric patients, and patients younger than ≤65 years, the leucocyte cut-off can be increased to 170/*μ*L.

## 1. Introduction

The prevalence and severity of urinary tract infections (UTI) depends on demographic characteristics, the clinical presentation, and the individual medical history [1, 2]. For instance, UTI is the second most prevalent infection seen in women aged 65 years or over [3], with a gradual increase with age [4]. In susceptible subgroups, such as immunosuppressed, diabetic, and geriatric patients, there is a greater risk of death and life-threatening complications such as urosepsis [3, 5, 6]. In diabetic patients, the mortality attributed to an infection is higher than that in the population without diabetes [7]. Thus, susceptible subgroups require special attention with respect to UTI.

The gold standard for the diagnosis of a UTI is still significant bacterial growth in urine culture combined with the clinical presentation of the patient [8]. However, the results of a urine culture can take up to several days—usually at least 24 hours—before they become available, and a prompt decision on treatment has to be taken within a shorter time frame. Thus, the decision for empirical antibiotic treatment is often based on clinical symptoms and more rapidly available laboratory tests, such as urine dipstick, microscopic examination, and, nowadays, urine flow cytometry [9]. All of these are considered useful tools for predicting significant bacterial growth in urine culture and are therefore used to diagnose a UTI.

Urine flow cytometry (UFC) has the advantage that it is standardized, less expensive than urine culture and microscopic examination, and rapidly available—especially in comparison to urine culture [10].

To rule out negative urine culture (nonsignificant bacterial growth), most studies have focused on fixed cut-offs determined by UFC for counts of leucocytes and bacteria in the urine (UFC leucocytes and UFC bacteria, respectively) that help to decide whether culturing is rational and indicated for treatment [11–13]. Several diagnostic algorithms suggest an “optimal” cut-off for predicting significant bacterial growth in urine culture [14]. Thus, the diagnosis of a UTI remains challenging, as the individual medical history and comorbidities of the patient might influence the diagnostic validity of fixed UFC cut-offs for predicting significant bacterial growth in urine culture. Only a few studies have evaluated the use of adjusted cut-offs for bacterium and leucocyte counts determined by UFC in specific clinical presentations—such as greater age, sex, and typical symptoms such as dysuria, renal insufficiency, diabetes, and other immunosuppression [1, 15–17].

The aim of this study is thus to develop, validate, and evaluate adapted cut-off values that result in a high negative predictive value for significant bacterial growth in urine culture in common and important clinical presentation subgroups—such as women, dysuric patients, diabetics, immunosuppressed patients, or patients younger than 65 years of age.

## 2. Methods

### 2.1. Study Design

This is a single-center, retrospective, observational study with data from patients of the emergency department (ED) of Bern University Hospital, Switzerland. More than 35,000 patients per year are treated at our ED. The patients' data were anonymized. The study was approved by the ethical committee of the canton of Berne (KEK: 2016-01298) and performed according to Swiss law. Individual informed consent was waived, as the data had been anonymized.

### 2.2. Study Population, Definition of Patient Clinical Presentation, and Data Extraction

This is a secondary analysis of a published data set, in which we developed generic tools to predict significant bacterial growth in urine culture from UFC measurements [14]. In the study presented here, we focus on patient characteristics: (i) age (>65 years and ≤65 years); (ii) gender; (iii) fever (>38.0°C on presentation in accordance with a previous UFC study [15]); (iv) urinary symptoms such as dysuria, urinary frequency, and UTI-specific abdominal pain, including suprapubic pain, lower abdominal pain, or flank pain; (v) renal insufficiency (classified by the medical history); (vi) diabetic patients; and (vii) immunosuppressed patients. Immunosuppression was defined as cancer under chemotherapy, transplantation, or a current hematological or rheumatological disease.

**Figure 1:**
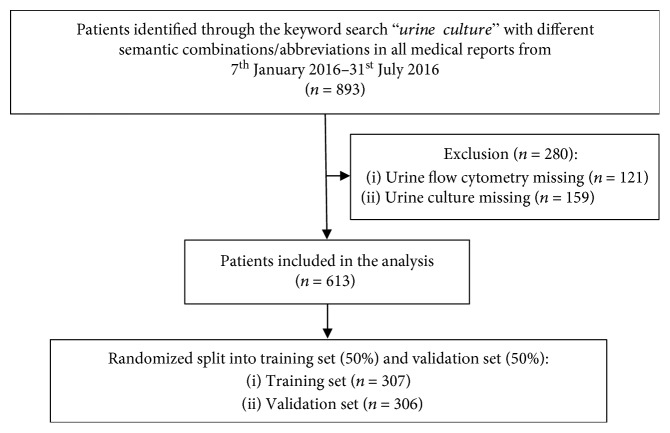
Flowchart.

The study population was obtained through a keyword search for “urine culture” in the medical file. The inclusion criteria were as follows: age > 16 years, presentation at the ED from January 7th, 2016, to July 31st, 2016, and urine culture and urine flow cytometry obtained within 24 hours of presentation. Thus, we included all patients for whom the attending physician decided to carry out urine flow cytometry and urine culture, as they were assumed to have suspected UTI. The flow chart and reasons for exclusion are shown in Figure 1. The medical history and clinical presentation are routinely recorded by the attending physician. The records were stored in our emergency department's medical database (E-Care, ED 2.1.3.0, Turnhout, Belgium).

### 2.3. Laboratory Analysis

Urine culture (gold standard) was obtained from a clean midstream/catheter urine at the ED and sent to the laboratory within two hours. After 24 h and 48 h of incubation, the results of the urine culture were recorded by the Department of Clinical Microbiology. Urine flow cytometry was performed by the Center of Laboratory Medicine at Bern University Hospital (the Inselspital), an ISO 17025-accredited laboratory. The UFC was performed with the UX-2000 flow cytometer (Sysmex Corporation, Kobe, Japan). The UFC is fully automated, and results can be viewed online by the attending physician 30 min after the start of the analysis. The procedures for urine collection, storage, and analysis are described in a previous publication [14].

### 2.4. Outcomes

Significant bacterial growth in the urine culture, defined as at least 10^4^ colony forming units (CFU) per mL, was taken as the gold standard.

### 2.5. Statistics

Statistical analysis was performed with Stata® 13.1 (StataCorp, College Station, Texas, USA).

To validate the obtained cut-offs, the data set was randomly divided into training and validation sets in a 1 : 1 ratio.

The different clinical presentations were separately analyzed as follows: First, the area (AUC) under a receiver operating curve (ROC) and its 95% confidence interval (CI) were calculated following logistic regression. This was then used to determine the discriminatory performance of UFC bacterium and UFC leucocyte counts for predicting significant bacterial growth in urine culture in the specific clinical presentation.

Second, the following adjusted cut-offs for bacterium and leucocyte counts in UFC were established for the training set:

- Cut-offs with the highest Youden's index (sensitivity + specificity − 1)
- Sensitivity-optimized LDA_95_ equations (test positive:⇔ln(UFC leucocytes + 1) > *a* × ln(UFC bacteria + 1)–*b*); the parameters *a* and *b* are determined through discriminant analysis and the optimization criterion that sensitivity should be above 0.95 in the training set (for easier reading and to derive a formula valid for all UFC values, the formula presented by Monsen and Ryden was slightly changed [18])
- Optimized sensitivity cut-offs defined as the highest bacterium or leucocyte count with a sensitivity ≥ 0.95; a test was defined as negative if both cut-offs are below the identified thresholds.

Third, these cut-offs were used in the validation sample to assess the diagnostic performance with respect to sensitivity, specificity, and positive and negative likelihood ratio (LR). The in-house cut-off for a positive test used at our hospital is UFC bacteria > 125/*μ*L or UFC leucocytes > 17/*μ*L; this was also validated. Last, the diagnostic performance of the different cut-offs in the clinical presentations was compared among the different methods.

## 3. Results

In total, 613 (100%) patients with a median age of 60 years (interquartile range: 46-75) were included in the analysis (Figure 1).

Table 1 shows that more than half of the study population was aged <65 years old (55.6%). Immunosuppression was found in 35.1%. Fever was present in 31.4%, while diabetes was coded in 22.2% and renal insufficiency in 33.9% of the patients. Typical symptoms for a UTI were less common, with 18.3% dysuria, 12.7% urinary frequency, and 19.4% with UTI-specific abdominal pain (including lower abdominal pain, suprapubic pain, and flank pain). Three hundred seven (307) consultations were randomized to the training set and 306 patients to the validation set. Significant bacterial growth in urine culture, defined as at least 10^4^ CFU/mL, was found in approximately four out of ten patients (40.2%). *E. coli* was identified in 48.6% of those patients.

The most common urological diagnosis was possible urogenital infection (29.5%). Other important discharge diagnoses were uncomplicated UTI (13.4%) and urosepsis (9.6%), as well as “no specific urological diagnosis” (25.6%, e.g., other infectious disease and respiratory problem).

### 3.1. Incidence of Significant Bacterial Growth in Urine Culture and Predictive Accuracy of UFC Bacterium and UFC Leucocyte Counts

The incidence of significant bacterial growth in urine culture was higher in females and older people (49.2% and 46.3%, respectively) than in males and younger people (32.3% and 35.8%, respectively). Depending on the clinical symptoms and comorbidities, the incidence ranged from 32.3% in male patients to 61.5% in patients with urinary frequency (Table 2). The incidence of significant bacterial growth in the urine culture in the different clinical presentations did not differ significantly between the training and validation sets (all *p* > 0.05).

The discriminatory accuracy values of UFC leucocyte and UFC bacterium counts to predict significant bacterial growth in urine culture measured by the AUC were at least 0.88 in all studied clinical presentations. In female patients, the AUC was 0.88 (95% CI: 0.85, 0.92) and in male patients 0.95 (95% CI: 0.92, 0.97). In the other clinical presentations, the 95% CI of the AUC overlapped, although the actual AUC values were slightly different (Table 2).

### 3.2. Adapted Cut-Offs with the Highest Youden's Index

The training sample was used to find adapted cut-offs for UFC bacterium and UFC leucocyte counts that maximized Youden's index in each clinical subgroup. Striking differences in the cut-offs were found (Table 3). For example, if Youden's cut-off was 74/*μ*L for UFC leucocyte counts and 63/*μ*L for UFC bacterium counts, Youden's index in patients with renal insufficiency was 0.5. In patients without diagnosed renal insufficiency, the Youden-based cut-off for UFC leucocyte counts was 108/*μ*L and for UFC bacterium counts 470/*μ*L, giving Youden's index of 0.7.

Maximal values of Youden's index were associated with high-sensitivity values, which were all above 88.2%, with lower sensitivity (<90%) in diabetic and female patients in the validation set. Specificity was lower, with a maximum of 88.3% (range: 46.2% in patients with UTI-specific abdominal complaints up to 88.3% in febrile patients). The high-sensitivity values were reflected by a small negative likelihood ratio (<0.1), indicating a large decrease in the posttest probability of significant bacterial growth, while the positive likelihood ratio was <5 in all clinical presentations (slight increase in posttest probability) [19].

### 3.3. Adapted Cut-Offs with Optimized Sensitivity > 95% Compared with the In-House Cut-Offs

Two different methods were used to adapt cut-offs to high sensitivity. The first approach was to use the adapted LDA_95_ equation. The second approach was to optimize sensitivity (>95%) for each group of patients—firstly for the UFC bacterium count and secondly for the UFC leucocyte count—and then to combine these cut-offs in a single test. A test was defined as positive if either the UFC leucocyte or the UFC bacterium cut-off was exceeded. These two approaches gave different results for diagnostic performance (Tables 4 and 5). When specificity was moderate, the adapted LDA_95_ equations failed to show good sensitivity (>90%) for several groups of patients, including male, older, and diabetic patients (Table 4).

For all clinical presentations, the in-house cut-offs (test positive if UFC leucocytes > 17/*μ*L or UFC bacteria > 125/*μ*L) exhibited high sensitivity and good negative likelihood ratios (<0.1). In total, 184 of the 613 patients (30.0%) had UFC leucocyte < 17/*μ*L and UFC bacteria < 125/*μ*L in the flow cytometry results. The adapted sensitivity cut-off values were similar to the in-house values. In female, younger, and dysuric patients, high sensitivity was obtained even with higher cut-offs for UFC leucocyte counts (169/*μ*L, 169/*μ*L, and 205/*μ*L) and a similar value for UFC bacterium counts (103/*μ*L). Specificity was low for all tested cut-offs—especially the in-house cut-off—and ranged from 22.2% in patients with urinary frequency to 60.6% in male patients (Table 5).

## 4. Discussion

This is the first study to focus on the development, evaluation, and validation of cut-off parameters for UFC bacterium and UFC leucocyte counts in different clinical scenarios in an emergency department population. This can then be used to predict significant bacterial growth in urine culture, which in turn indicates a UTI and thus the necessity of antibiotic treatment. The study covered patients of different sex, age, symptoms (UTI-specific abdominal pain, fever, urinary frequency, and dysuria), and comorbidities (diabetes, renal insufficiency, and immunosuppression).

In all studied clinical presentations, the discriminative accuracy of UFC leucocyte and UFC bacterium counts for significant bacterial growth in urine culture was good (AUC ≥ 0.88). With the in-house cut-offs, a test was defined as positive if UFC leucocyte counts were >17/*μ*L or UFC bacterium counts > 125/*μ*L and with this exhibited high sensitivity in all groups of patients. Compared with the cut-offs for optimized sensitivity of >95% in female, younger, and dysuric patients, high sensitivity and even higher specificity were obtained with higher cut-offs for UFC leucocyte counts (169/*μ*L, 169/*μ*L, and 205/*μ*L).

Although this is sometimes neglected in clinical practice, the posttest probability of a disease depends on the incidence of a disease in the specific clinical setting and the likelihood ratio (LR) of the test [20]. Given a specific clinical presentation with a specific symptom *A*, the pretest probability, the incidence of the disease, can be converted to the odds for the disease. The odds multiplied by the LR_A_(+) of the disease in the specific presentation with symptom *A* and the general LR_Test_(+) leads to the posttest odds, which can be transformed to the posttest probability [19]. Posttest probability is of great help in the in-depth interpretation of test results [21]. However, this construct is not of great use in clinical practice, as it requires the specific LR(+) for each symptom as well as for symptom combinations and demands complex calculations [19]. Nevertheless, it is important that clinicians understand the primary concept of the pre- and the posttest probability. An alternative and more useful concept is the use of adapted cut-offs for diagnostic tests, which are, for instance, used in the diagnosis of venous embolism with age-adjusted D-dimers [22]. To rule out a diagnosis, it is necessary that the adapted cut-offs consider the possible higher or lower incidence of the disease in specific subgroups; otherwise, the test may not be valid in the specific presentation.

In our study, the incidence of significant bacterial growth in urine culture ranged from 32.8% in febrile patients to 61.5% in patients with urinary frequency (Table 2). These figures are comparable to the incidence of significant bacterial growth in urine culture in other studies [23]. The relatively low incidence of significant bacterial growth in urine cultures in immunosuppressed and febrile patients may be explained by the relatively broad indication for obtaining a urine culture in these patients, even when there is no great suspicion of a urological infection.

As suggested by different studies [20, 24, 25], one strategy for establishing cut-off values is to maximize Youden's index. Our results suggest that this strategy does not lead to useful cut-offs in many clinical presentations, as the resulting cut-offs can neither be used to “rule in” (high LR(+)) nor be used to “rule out” (LR(-)) significant bacterial growth in the urine culture but lead to balanced cut-offs with regard to LR(+)/LR(-). In general, cut-offs established by maximizing Youden's index should be treated cautiously, and the detailed diagnostic performance of such tests should be demonstrated. More adequate approaches to establish “optimal” cut-offs require estimating the “costs” of misdiagnoses, false positives, and false negatives [26], a task which is hard to accomplish. Thus, we focused on ruling out the diagnosis in the further analyses by maximizing sensitivity. The suggested linear discriminant analysis, LDA_95_ [18], is not very feasible in everyday use, as a calculator or computer is needed to evaluate the results of a urine culture and the results found in this study were not convincing. We slightly modified the formula presented by Monsen and Ryden [18], in order to assure that the formula was valid for all numbers of urine flow bacterium and leucocyte counts; this had no impact on the outcome. Adapted cut-offs for the clinical presentations were therefore developed by maximizing the sensitivity of both UFC leucocyte and bacterium counts and combining these into one test. In the setting of immunosuppression, for instance, lower cut-offs for UFC bacterium and UFC leucocyte counts were found to lead to high sensitivity (>95%). Stefanovic et al. also studied the diagnostic performance of urine flow cytometry in immunosuppressed patients. For high sensitivity, they employed even lower cut-offs for UFC bacterium counts ≥ 20/*μ*L or UFC leucocyte counts > 5/*μ*L [17]. Even with this low cut-off, the sensitivity was lower (90.6%) for immunosuppressed patients than for nonimmunosuppressed patients. This is in contrast to our study, in which the higher cut-off was sufficient to rule out significant bacterial growth in urine culture. In another study, the use of age-specific UFC parameters was recommended [16]; it was found that for people aged ≥65 years, a single cut-off of UFC bacterium counts of 200/*μ*L leads to very high sensitivity and high specificity (99.1% and 91.6%, respectively) with a minimal rate of 0.87% false negative results.

Manoni et al. suggested using UFC leucocytes > 17/*μ*L or UFC bacteria > 125/*μ*L to define a pathological test [12]. Our study shows that these cut-offs (Table 5, the in-house cut-offs) are valid for ruling out significant bacterial growth in urine culture in all studied clinical presentations; this cut-off is therefore recommended as a safe rule-out cut-off in the ED. With this high-sensitivity cut-off, as many as 30% of the urine cultures could have been avoided in our study. Martín-Gutiérrez et al. showed that an even higher cut-off of 200/*μ*L for the UFC bacterium count could help to avoid the culture of up to 60.2% of samples in the elderly population (>65 years) [16].

However, with these cut-offs, specificity was lacking, and in some groups, such as females, people aged ≤65 years, and dysuric patients, even higher cut-offs for UFC leucocyte counts (169/*μ*L, 169/*μ*L, and 205/*μ*L, respectively) can assure high sensitivity.

### 4.1. Strengths and Limitations

Our results were obtained with the UX-2000. However, as the UX-2000 series has the same technology as the frequently used UF-1000i series to measure urine leucocyte and bacterium counts; our cut-offs can also be applied to the latter machines.

In contrast to many other studies that focused on UFC cut-off values, a particular strength of this study lies in the external validity of our cohort. In their systematic review, Shang et al. concluded that most studies were laboratory based. Thus, it was unclear whether they were generalizable to clinically suspected UTI [13]. On the other hand, our data were collected in an ED population, and the results may not be transferred to a non-ED patient population, such as a health care center.

Furthermore, trials are recommended to evaluate the use of clinically adapted cut-offs to determine the economic benefit, as well as to quantify the impact of adapted cut-offs on the prescription of (unnecessary) antibiotics.

The greatest limitation of our study is the retrospective design. While the quality of the extracted data can be assured for both the exposure (UFC data) and the outcome data (significant bacterial growth in urine culture), as they are hard outcomes measured in certified laboratories, the evaluated clinical presentations were predominantly defined through the electronic medical record. Thus, information and misclassification bias through incomplete documentation is an important potential limitation of this study. The number of patients in some subgroups may have been too small to detect differences in the predictive accuracy in some specific presentations (potentially underpowered statistics); this is reflected in the wide confidence intervals. Furthermore, one particularly important subgroup of patients could not be studied with our retrospective study sample, i.e., patients with a high suspicion of a UTI, namely, patients with more than one symptom, for example, dysuria, fever, and suprapubic pain. As the sample size of this study was small, we suggest that our results should be verified in larger, prospective studies. As a consequence of the small sample size, one cannot combine the results and apply them to patients who have a combination of the different attributes for which criteria were presented in this study. In general, if more than one applicable category with adapted cut-off exists, we recommend using the one with the lowest cut-off for a safe exclusion of future significant bacterial growth in urine culture. Prospective studies are needed to address the impact of the diagnostic accuracy for significant bacterial growth in urine culture for UFC parameters in the setting in which the clinician greatly suspects a UTI. Moreover, our design only enabled us to include people for whom a urine culture was obtained in ordinary clinical practice. In a prospective design, a urine culture would have been obtained for all patients with a UFC. As the patients from the examined presentations overlap, the results are not independent and cannot be combined to calculate (for instance) the positive likelihood ratio to rule out significant bacterial growth in urine culture in an immunosuppressed, female, older patient.

## 5. Conclusions

Clinicians should be aware that in specific patient groups, e.g., in females, the predictive accuracy of UFC leucocyte and UFC bacterium counts to predict significant bacterial growth in urine culture is diminished and that the predictive accuracy of cut-offs depends on the clinical setting. On the basis of our findings, UFC can be used to rule out significant bacterial growth in urine culture with high sensitivity by the use of the cut-offs for UFC bacterium counts ≤ 125/*μ*L and UFC leucocyte counts ≤ 17/*μ*L—independently of the clinical presentation, sex, age, or comorbidities such as diabetes, renal insufficiency, and immunosuppression. Even higher UFC leucocyte cut-offs (169/*μ*L, 169/*μ*L, and 205/*μ*L) are justified in females, people aged ≤65 years, and dysuric patients.

## Figures and Tables

**Table 1:** Patient characteristics (*n* = 613).

Characteristics (*n* (%))	Total (*n* = 613)		Training set (*n* = 307)		Validation set (*n* = 306)	
*Sex*						
Male	316	(51.5)	157	(51.1)	159	(52.0)
Female	297	(48.5)	150	(48.9)	147	(48.0)
*Age*						
>65 years	272	(44.4)	130	(42.4)	142	(46.4)
≤65 years	341	(55.6)	177	(57.6)	164	(53.6)
*Clinical presentation*						
Dysuria	112	(18.3)	56	(18.2)	56	(18.3)
Urinary frequency	78	(12.7)	40	(13.0)	38	(12.4)
Fever (>38.0°C)^1^	192	(31.4)	96	(31.4)	96	(31.5)
Urinary tract infection-specific abdominal pain^2^	119	(19.4)	61	(19.9)	58	(19.0)
*Comorbidities*						
Diabetes mellitus	136	(22.2)	62	(20.2)	74	(24.2)
Renal insufficiency	208	(33.9)	112	(36.5)	96	(31.4)
Immunosuppression	215	(35.1)	105	(34.2)	110	(36.0)
Hospitalisation	451	(73.6)	225	(73.3)	226	(73.9)

^1^Two values (one from the training and one from the validation set) were missing; thus, *n* = 611. ^2^This includes lower abdominal, suprapubic, and flank pain.

**Table 2:** Discriminatory accuracy of UFC leucocyte and UFC bacterium counts measured by the area under the receiver operating curve of the logistic regression to predict significant bacterial growth in urine culture.

	Total (*n* = 613) SBU (%)	AUC (95% CI)	Training set (*n* = 307) SBU (%)	Validation set (*n* = 306) SBU (%)
*Sex*				
Male	32.3	0.95 (0.92, 0.97)	29.9	34.6
Female	49.2	0.88 (0.85, 0.92)	48.7	49.7
*Age*				
>65 years	46.3	0.95 (0.92, 0.97)	45.4	47.2
≤65 years	35.8	0.90 (0.86, 0.93)	34.5	37.2
*Dysuria*				
Yes	54.5	0.88 (0.82, 0.94)	57.1	51.8
No	37.3	0.92 (0.90, 0.95)	35.1	39.6
*Urinary frequency*				
Yes	61.5	0.88 (0.80, 0.96)	70.0	52.6
No	37.4	0.92 (0.90, 0.94)	34.5	40.3
*Fever (>38.0°C)^1^*				
Yes	32.8	0.94 (0.90, 0.98)	28.1	37.5
No	43.9	0.91 (0.88, 0.94)	44.3	43.5
*UTI-specific abdominal pain^2^*				
Yes	54.6	0.88 (0.81, 0.93)	54.1	55.2
No	37.0	0.92 (0.90, 0.95)	35.5	38.7
*Diabetes*				
Yes	44.1	0.94 (0.90, 0.98)	41.9	46.0
No	39.4	0.91 (0.89, 0.94)	38.4	40.5
*Renal insufficiency*				
Yes	39.9	0.93 (0.89, 0.96)	37.5	42.7
No	40.7	0.92 (0.89, 0.94)	40.0	41.4
*Immunosuppression*				
Yes	33.5	0.92 (0.88, 0.96)	33.3	33.6
No	44.2	0.91 (0.89, 0.94)	42.1	46.4
*Total*	40.2	—	39.1	41.8

^1^Two values (one of training and one of the validation set) were missing; thus, *n* = 611. ^2^This includes lower abdominal, suprapubic, and flank pain. Abbreviations: AUC: area under the receiver operating curve; CI: confidence interval; SBU: significant bacterial growth in urine culture; UFC: urine flow cytometry; UTI: urinary tract infection.

**Table 3:** Established cut-off values for UFC bacterium and leucocyte counts per *μ*L with the highest Youden's index and the corresponding diagnostic performance in the validation sample in predicting significant bacterial growth in urine culture in different clinical scenarios.

Subgroup (no. in validation set)	Highest Youden's index		Diagnostic performance using the established cut-offs^∗^			
	Lc (/*μ*L)	Bact (/*μ*L)	Sensitivity (%) (95% CI)	Specificity (%) (95% CI)	LR(+)	LR(-)
*Sex*						
Male (*n* = 159)	74	71	94.5 (84.9, 98.9)	71.2 (61.4, 79.6)	3.28	0.08
Female (*n* = 147)	169	2883	89 (79.5, 95.1)	79.7 (68.8, 88.2)	4.39	0.14
*Age*						
>65 (*n* = 142)	57	63	95.5 (87.5, 99.1)	60.0 (48.0, 71.1)	2.39	0.07
≤65 (*n* = 164)	169	150	96.7 (88.7, 99.6)	64.1 (54.0, 73.3)	2.69	0.05
*Dysuria*						
Yes (*n* = 56)	205	87	96.6 (82.2, 99.9)	66.7 (46.0, 83.5)	2.90	0.05
No (*n* = 250)	74	441	93.3 (87.3, 97.7)	74.8 (67.1, 81.5)	3.73	0.08
*Urinary frequency*						
Yes (*n* = 38)	108	409	100 (83.2, 100)	55.6 (30.8, 78.5)	2.25	—
No (*n* = 268)	74	83	96.3 (90.8, 99)	60.6 (52.6, 68.2)	2.45	0.06
*Fever (>38.0°C)^1^*						
Yes (*n* = 96)	108	527	91.7 (77.5, 98.2)	88.3 (77.4, 95.2)	7.86	0.09
No (*n* = 209)	171	99	94.5 (87.6, 98.2)	61.0 (51.6, 69.9)	2.42	0.09
*UTI-specific abdominal pain^2^*						
Yes (*n* = 58)	169	128	96.9 (83.8, 99.9)	46.2 (26.6, 66.6)	1.80	0.07
No (*n* = 248)	69	83	95.8 (89.7, 98.9)	59.9 (51.6, 67.7)	2.40	0.07
*Diabetes*						
Yes (*n* = 74)	76	1061	88.2 (72.5, 96.7)	77.5 (61.5, 89.2)	3.92	0.15
No (*n* = 232)	137	441	96.8 (91.0, 99.3)	77.5 (69.7, 84.2)	4.31	0.04
*Renal insufficiency*						
Yes (*n* = 96)	74	63	95.1 (83.5, 99.4)	56.4 (42.3, 69.7)	2.18	0.08
No (*n* = 210)	108	470	94.3 (87.1, 98.1)	76.4 (67.9, 83.6)	4.00	0.08
*Immunosuppression*						
Yes (*n* = 110)	74	127	97.3 (85.8, 99.9)	72.6 (60.9, 82.4)	3.55	0.04
No (*n* = 196)	151	470	94.5 (87.6, 98.2)	74.3 (64.8, 82.3)	3.68	0.07

^1^One patient in the validation set did not have sufficient data to determine if temperature > 38.0°C. ^2^This includes lower abdominal, suprapubic, and flank pain. Abbreviations: AUC: area under the receiver operating curve; Bact: UFC bacterium counts; CI: confidence interval; Lc: UFC leucocyte counts; LR: likelihood ratio; SBU: significant bacterial growth in urine culture; UFC: urine flow cytometry; UTI: urinary tract infection.

**Table 4:** Established high-sensitivity test with the use of an adapted 95 percent linear discrimination analysis equation (LDA_95_) and its validation. LDA_95_ equation: ln(UFC leucocyte + 1) > *A* × ln(UFC bacteria + 1) + *B*.

Subgroup	LDA_95_-equat. coefficients		Diagnostic performance using the established cut-offs^∗^			
	*A*	*B*	Sensitivity (%) (95% CI)	Specificity (%) (95% CI)	LR(+)	LR(-)
*Sex*						
Male (*n* = 159)	-4.6	21.2	89.1 (77.8, 95.9)	76.0 (66.6, 83.8)	3.71	0.14
Female (*n* = 147)	-3.4	18.9	95.9 (88.5, 99.1)	51.4 (39.4, 63.1)	1.97	0.08
*Age*						
>65 (*n* = 142)	-20.4	86.8	89.6 (79.7, 95.7)	70.7 (59.0, 80.6)	3.05	0.15
≤65 (*n* = 164)	-1.6	10.5	95.1 (86.3, 99.0)	63.1 (53.0, 72.4)	2.58	0.08
*Dysuria*						
Yes (*n* = 56)	-1.4	10.4	96.6 (82.2, 99.9)	66.7 (46.0, 83.5)	2.90	0.05
No (*n* = 250)	-4.5	20.8	91.9 (84.7, 96.4)	58.3 (50.0, 66.2)	2.20	0.14
*Urinary frequency*						
Yes (*n* = 38)	-2.3	17.8	100 (83.2, 100)	66.7 (41.0, 86.7)	3.00	—
No (*n* = 268)	-4.2	19.7	90.7 (83.6, 95.5)	62.5 (54.5, 70.0)	2.42	0.15
*Fever (>38.0°C)^1^*						
Yes (*n* = 96)	-1.3	8.2	94.4 (81.3, 99.3)	68.3 (55.0, 79.7)	2.98	0.08
No (*n* = 209)	-4.9	25.0	92.3 (84.8, 96.9)	66.1 (56.8, 74.6)	2.72	0.12
*UTI-specific abdominal pain^2^*						
Yes (*n* = 58)	-1.8	14.0	96.9 (83.8, 99.9)	57.7 (36.9, 76.6)	2.29	0.05
No (*n* = 248)	-4.5	20.5	91.7 (84.2, 96.3)	63.8 (55.6, 71.4)	2.53	0.10
*Diabetes*						
Yes (*n* = 74)	-4.2	25.0	79.4 (62.1, 91.3)	80.0 (64.4, 90.9)	3.97	0.26
No (*n* = 232)	-3.0	15.3	94.7 (88.0, 98.3)	61.6 (52.9, 69.7)	2.47	0.09
*Renal insufficiency*						
Yes (*n* = 96)	-2.4	13.0	87.8 (73.8, 95.9)	67.3 (54.3, 95.9)	2.68	0.18
No (*n* = 210)	-3.9	21.0	93.1 (85.6, 97.4)	69.9 (61.0, 77.9)	3.10	0.10
*Immunosuppression*						
Yes (*n* = 110)	-1.0	6.5	97.3 (85.8, 99.9)	64.4 (52.3, 75.3)	2.73	0.04
No (*n* = 196)	-5.9	31.2	91.2 (83.4, 96.1)	67.6 (57.8, 76.4)	2.82	0.13

^1^One patient in the validation set did not have sufficient data to determine if temperature > 38.0°C. ^2^This includes lower abdominal, suprapubic, and flank pain. Abbreviations: CI: confidence interval; LDA: linear discrimination analysis; LR: likelihood ratio; UTI: urinary tract infection.

**Table 5:** Diagnostic performance of the in-house cut-offs and the sensitivity-optimized cut-offs for each clinical scenario.

Subgroup	Diagnostic performance using in-house cut-off (UFC leucocyte > 17/*μ*L, UFC bacteria > 125/*μ*L)				Optimized cut-off corresponding diagnostic performance^∗^					
	Sensitivity (%) (95% CI)	Specificity (%) (95% CI)	LR(+)	LR(-)	Cut-offs (/*μ*L)		Sensitivity (%) (95% CI)	Specificity (%) (95% CI)	LR(+)	LR(-)
					Lc	Bact				
*Sex*										
Male (*n* = 159)	96.4 (87.5, 99.6)	60.6 (50.5, 70.0)	2.44	0.06	74	17	100 (93.5, 100)	54.8 (44.7, 64.6)	2.21	—
Female (*n* = 147)	98.6 (92.6, 100)	37.8 (26.8, 49.9)	1.59	0.04	169	103	97.3 (90.5, 99.7)	45.9 (34.3, 57.9)	1.8	0.06
*Age*										
>65 (*n* = 142)	95.5 (87.5, 99.1)	58.7 (46.7, 69.9)	2.31	0.07	57	21	100 (94.6, 100)	50.7 (38.9, 62.4)	2.03	—
≤65 (*n* = 164)	100 (94.1, 100.0)	45.6 (35.8, 55.7)	1.84	—	169	103	96.7 (88.7, 99.6)	60.2 (50.1, 69.7)	2.43	0.05
*Dysuria*										
Yes (*n* = 56)	100 (88.1, 100)	33.3 (16.5, 54.0)	1.5	—	205	103	96.6 (82.2, 99.9)	66.7 (46.0, 83.5)	2.90	0.05
No (*n* = 250)	97 (91.4, 99.4)	54.3 (46, 62.4)	2.12	0.06	74	21	100 (96.3, 100)	45.7 (37.6, 54)	1.84	—
*Urinary frequency*										
Yes (*n* = 38)	100 (83.2, 100)	22.2 (6.4, 47.6)	1.29	—	108	210	100 (83.2, 100)	50.0 (26.0, 74.0)	2.00	—
No (*n* = 268)	97.2 (92.1, 99.4)	54.4 (46.3, 62.3)	2.13	0.05	74	21	100 (96.6, 100)	47.5 (39.6, 55.5)	1.90	—
*Fever (>38.0°C)^1^*										
Yes (*n* = 96)	97.2 (85.5, 99.9)	60.0 (46.5, 72.4)	2.43	0.05	108	14	100 (90.3, 100)	48.3 (35.2, 61.6)	1.94	—
No (*n* = 209)	97.8 (92.3, 99.7)	46.6 (37.4, 56.0)	1.83	0.05	171	22	96.7 (90.7, 99.3)	48.3 (39.0, 57.7)	1.87	0.07
*UTI-specific abdominal pain^2^*										
Yes (*n* = 58)	100 (89.1, 100)	34.6 (17.2, 55.7)	1.53	—	169	209	96.9 (83.8, 99.9)	53.8 (33.4, 73.4)	2.10	0.06
No (*n* = 248)	96.9 (91.1, 99.4)	53.9 (45.7, 62.1)	2.10	0.06	69	21	100 (96.2, 100)	47.4 (39.2, 55.6)	1.90	—
*Diabetes*										
Yes (*n* = 74)	97.1 (84.7, 99.9)	57.5 (40.9, 73)	2.28	0.05	76	17	100 (89.7, 100)	50.0 (33.8, 66.2)	2.00	—
No (*n* = 232)	97.9 (92.5, 99.7)	49.3 (40.7, 57.9)	1.93	0.04	137	56	97.9 (92.5, 99.7)	55.8 (47.1, 64.2)	2.21	0.04
*Renal insufficiency*										
Yes (*n* = 96)	95.1 (83.5, 99.4)	45.5 (32.0, 59.4)	1.74	0.11	74	21	100 (91.4, 100)	49.1 (35.4, 62.9)	2.00	—
No (*n* = 210)	98.9 (93.8, 100)	53.7 (44.4, 62.7)	2.13	0.02	108	54	97.7 (91.9, 99.7)	54.5 (45.2, 63.5)	2.15	0.04
*Immunosuppression*										
Yes (n = 110)	100 (90.5, 100)	57.5 (45.4, 69.0)	2.35	—	74	24	97.3 (85.8, 99.9)	53.4 (41.4, 65.2)	2.09	0.05
No (*n* = 196)	96.7 (90.7, 99.3)	46.7 (36.9, 56.7)	1.81	0.07	151	21	97.8 (92.3, 99.7)	46.7 (36.9, 56.7)	1.83	0.05

^1^One patient in the validation set did not have sufficient data to determine if temperature > 38.0°C. ^2^This includes lower abdominal, suprapubic, and flank pain. Abbreviations: Bact: UFC bacterium counts; CI: confidence interval; Lc: UFC leucocyte counts; LR: likelihood ratio; UFC: urine flow cytometry; UTI: urinary tract infection.

## Data Availability

The statistical data used to support the findings of this study are available from the corresponding author upon request.
